# Comparative docking analysis of cholesterol analogs to ion channels to discriminate between stereospecific binding vs. stereospecific response

**DOI:** 10.1080/19336950.2019.1606670

**Published:** 2019-04-29

**Authors:** Nicolas A. Barbera, Baruch Minke, Irena Levitan

**Affiliations:** a Division of Pulmonary, Critical Care, Sleep and Allergy, Department of Medicine, University of Illinois at Chicago, Chicago, IL, USA; b Department of Chemical Engineering, University of Illinois at Chicago, Chicago, USA; c Department of Medical Neurobiology, and the Edmond and Lily Safra Center for Brain Sciences (ELSC), Faculty of Medicine, the Hebrew University, Jerusalem, Israel

**Keywords:** Inwardly rectifying K+ (Kir) channels, Transient Receptor Potential (TRP) channels, cholesterol, epicholesterol, ent-cholesterol

## Abstract

Cholesterol is a major component of the membrane and a key regulator of many ion channels. Multiple studies showed that cholesterol regulates ion channels in a stereospecific manner, with cholesterol but not its chiral isomers having a functional effect. This stereospecificity has been universally attributed to the specificity of cholesterol binding, with the assumption that only native cholesterol binds to the channels whereas its isomers do not. In this study, we challenge this paradigm by docking analyses of cholesterol and its chiral isomers to five ion channels whose response to cholesterol was shown to be stereospecific, Kir2.2, KirBac1.1, TRPV1, GABA_A_ and BK. The analysis is performed using AutoDock Vina to predict the binding poses and energies of the sterols to the channels and identify amino acids interacting with the sterol molecules. We found that for every ion channel tested herein all three sterols showed similar binding poses and significant overlap in the set of the amino acids that comprise the predicted binding sites, along with similar energetic favorability to these overlapping sites. We also found, however, that specific orientations of the three sterols within the binding sites of the channels are distinct, so that a subset of the interacting amino acids is unique to each sterol. We propose therefore, that contrary to previous thought, stereospecific effects of cholesterol should be attributed not to the lack of binding of the stereoisomers but to specific, unique interactions between the cholesterol molecule and the residues within the binding sites of the channels.

## Introduction

Cholesterol is an integral component of cell membranes, comprising anywhere from 10 to 45% of the lipid bilayer of mammalian cells [1]. In addition to playing an important role in controlling membrane fluidity and altering lipid packing [1,2], cholesterol has been shown to be an important regulator for a wide array of membrane-embedded proteins, including ion channels. For many of these channels, in particular K^+^ channels, cholesterol has an inhibitory effect on the channel activity. For example, inwardly rectifying K^+^ channels [3,4], Ca^2+-^sensitive K^+^ channels [5,6], and voltage-gated K^+^ channels [7,8] are all suppressed by the elevation of membrane cholesterol level. Likewise, voltage-gated Na^+^ and Ca^2+^ channels [9] and volume-regulated anion channels [10] are also negatively regulated by cholesterol. In contrast, cholesterol has been shown to be necessary for the activity of nicotinic acetylcholine receptors, and is thought to stabilize them in the open state [11,12,13].

A major tool in elucidating the mechanism of cholesterol-mediated regulation of ion channels involves a comparative analysis of different sterols, particularly chiral isomers of cholesterol. Chirality is an important structural feature of many organic molecules, and in part arises due to the tendency of carbon atoms to form bonds in tetrahedral arrangements. A consequence of this arrangement is that even relatively simply chemical compounds with identical chemical formulas can adopt structurally distinct geometries, called stereoisomers. Significantly, these geometries cannot be superimposed on one another, meaning that these stereoisomers are effectively separate molecules with distinct physiochemical properties from one another. The general assumption of this comparative method in most studies has been that chiral isomers of cholesterol cannot bind to cholesterol-binding sites and thus, if the effect of cholesterol was found to be stereo-specific, it should mean regulation via specific binding [3,4,14–18]. However, our recent work with Kir channels has challenged this assumption. Previously, we found that replacing 50% of cholesterol with epicholesterol not only abrogated the effects of cholesterol inhibition, but also appeared to increase channel activity, suggesting that epicholesterol acts as a competitive inhibitor of cholesterol [4]. Furthermore, we also found evidence of competitive inhibition between cholesterol and epicholesterol in KirBac1.1, a bacterial homologue of Kir channels [19]. This led us to consider an alternative hypothesis: cholesterol, and its optical isomers can in fact all directly interact with the channel, but the regulatory effect of cholesterol is stereospecific and is contingent on the specific structural arrangement of cholesterol molecules, as opposed to its chiral isomers. To test this, we performed docking analyses of cholesterol and two of its optical isomers, epicholesterol and ent-cholesterol on Kir2.2. We found that cholesterol, epicholesterol, and ent-cholesterol were all predicted to interact within the same site on the channel but with subtle differences in orientation due to the specific arrangements of each sterol’s chiral centers [20]. These subtle differences also mean differences in the residues with which each sterol interacts, and point to a way through which cholesterol modulates channel activity while its chiral isomers do not.

In the current study, we expand this idea to ion channels more broadly. We investigated and compared the predicted binding poses and energies of five different types of ion channels, Kir2.2, KirBac1.1, TRPV1, GABA_A_, and BK channels. Each of these channels has been experimentally tested with cholesterol and at least one of its chiral isomers epicholesterol or ent-cholesterol and found to be stereoselective [3,4,14,15,17,18,21]. We find that the predicted binding sites of cholesterol and its chiral isomers show overlap in their orientation and binding location for all the channels tested.

## Materials and methods

Docking analyses of cholesterol and its isomers to several types of ion channels were performed using AutoDock Vina [22]. Docking analyses were run with an exhaustiveness of 40. For each protein, the search space was defined such that the center of the search space aligned with the center of the transmembrane region of the protein and the z-axis boundaries set at the lipid-water interface, as defined by the OPM database [23]. Likewise, the designated search spaces covered regions on each channel encompassing both a single subunit and inter-subunit space. An example of a configuration file used in a docking analysis is shown in Suppl. Figure 1. For each docking analysis, the top scoring pose was examined. The RMSD and estimated binding energies of predicted binding poses were compared between each of the resultant 9 top poses for the three separate runs of cholesterol, epicholesterol, and ent-cholesterol, to determine similarity and reproducibility of the top pose. Interacting residues were determined to be those residues within 4.5Å of the predicted binding pose.

### Validation of cholesterol docking procedure using a solved crystal structure of a protein with bound cholesterol

**Figure 1.**
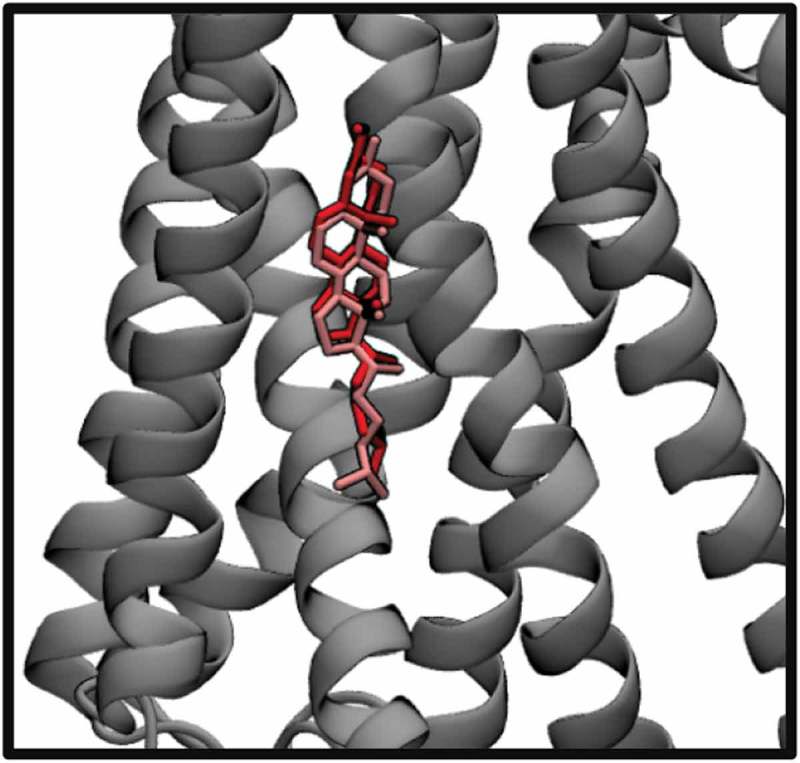
Predicted docking pose of cholesterol on the β2-adrenergic receptor, overlaid on top the crystal structure position of bound cholesterol.

The crystal structure for the β2-adrenergic receptor with cholesterol was taken from the PDB databank (PDB: 5D6L). All solvents and ligands were removed from the structure file, and 3 separate docking analyses were performed with cholesterol on the TM region, with x- and y- dimensions of 38.25 Å and 37.5 Å. We found that the top-scoring predicted binding pose for cholesterol shows good agreement with the crystal structure binding pose, with an RMSD of 3.83 Å (Figure 1). For both the crystal structure and the predicted pose, the smooth face of the cholesterol molecules is oriented toward the receptor, with the methyl groups facing toward the membrane environment.

*Structures for Kir2.2, KirBac1.1, TRPV1, GABA_A_, and BK* were taken from the PDB databank (PDB IDs: 3SPI, 1P7B, 3J9J, 4COF, 5TJ6, respectively). The crystal structure resolution of each structure is shown in Table 1. The x- and y-axis dimensions were defined in each case to encompass a single subunit. Specifically, the dimensions for each system were: 32.25 Å x 33 Å x 42.75 Å (Kir2.2), 46.5 Å x 33 Å x 36.75 Å (KirBac1.1), 47.25 Å x 47.25 Å x 33.75 Å (TRPV1), 29.25 Å x 45 Å x 47.25 Å (GABA_A_), and 42.75 Å x 42.75 Å x 42.75 Å (BK). For each ligand, three separate docking analyses were run to ensure reproducibility.

**Table 1.** Crystal structure resolutions.

Structure	PBD	Resolution
Kir2.2	3SPI	3.307 Å
KirBac1.1	1P7B	3.65 Å
TRPV1	3J9J	3.275 Å
GABA_A_	4COF	2.97 Å
BK	5TJ6	3.5 Å
β2AR	5D6L	3.2 Å

## Results

### Stereoisomers interact similarly in Kir2.2 and KirBac1.1

**Figure 2.**
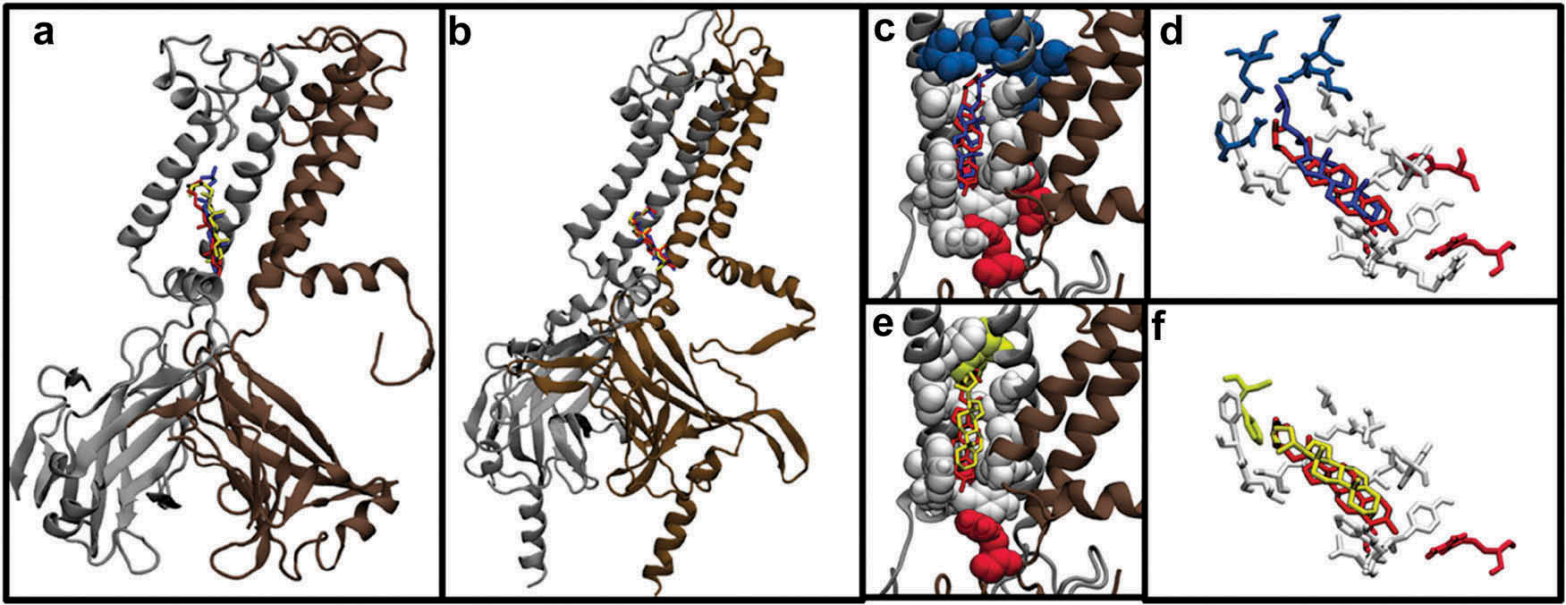
(a) Predicted binding poses of cholesterol, epicholesterol, and ent-cholesterol on the KirBac1.1 channel. (b) Predicted binding poses of cholesterol, epicholesterol, and ent-cholesterol on the Kir2.2 Channel. (c) Comparison of the binding poses of cholesterol and ent-cholesterol. (d) Close-up of the specific residues predicted to interact with cholesterol and ent-cholesterol. (e) Comparison of the binding poses of cholesterol and epicholesterol. (f) Close-up of the specific residues predicted to interact with cholesterol and epicholesterol.

As discussed previously, we found for Kir2.2 that the predicted binding sites for cholesterol, epicholesterol, and ent-cholesterol showed a high degree of overlap. Here, we extended the analysis to KirBac1.1, another inwardly rectifying potassium channel with a resolved crystal structure. Three sets of docking analyses were performed for cholesterol, epicholesterol, and ent-cholesterol on the transmembrane region of KirBac1.1. For each sterol, the predicted energies for the top scoring poses were −9.8, −9.4, and −10 kcal/mol, respectively, which is similar to previously reported binding energies for Kir2.2 (−8.5, −8.8, and −8.3 kcal/mol). The predicted binding poses of the cholesterol isomers in KirBac1.1 were structurally similar to one another, with the RMSD difference between the poses of cholesterol and epicholesterol in KirBac1.1 equal to 3.93 Å and the RMSD difference between cholesterol and ent-cholesterol equal to 3.82 Å. This similarity in binding poses and binding energies is also reflected visually in the predicted locations of the sterol-binding sites. As can be seen in Figure 2(a), all three sterols are predicted to occupy the same pocket within the transmembrane region of the channel, located on the inner-leaflet side. Furthermore, this is analogous to what we found previously with Kir2.2 channels, wherein the sterols are oriented with their hydroxyl groups facing the cytosolic side of the membrane and adjacent to the slide helix (Figure 2(b)). As was the case with Kir2.2, with KirBac1.1, the predicted binding poses of cholesterol, epicholesterol, and ent-cholesterol show significant overlap in the interacting residues, but with residues unique to each isomer. Specifically: all three sterols are predicted to interact with Trp^48^, Leu^51^, Tyr^52^, Trp^60^, Leu^67^, Leu^70^, Phe^71^, Gly^137^, Leu^140^, Ser^141^, and Leu^144^ (Figure 2(c-f)). Cholesterol, in part due to the orientation of its hydroxyl group, also uniquely interacts with residues Ala^55^ and Arg^153^, while epicholesterol uniquely interacts with Phe^132^, and ent-cholesterol interacts with Ala^109^, Gly^134^, Met^135^, and Ile^138^. The overlap of identified residues can be seen in the Venn diagram in **Suppl. Fig. 2A**.

### An overlap of cholesterol stereoisomers interacting in TRPV1

**Figure 3.**
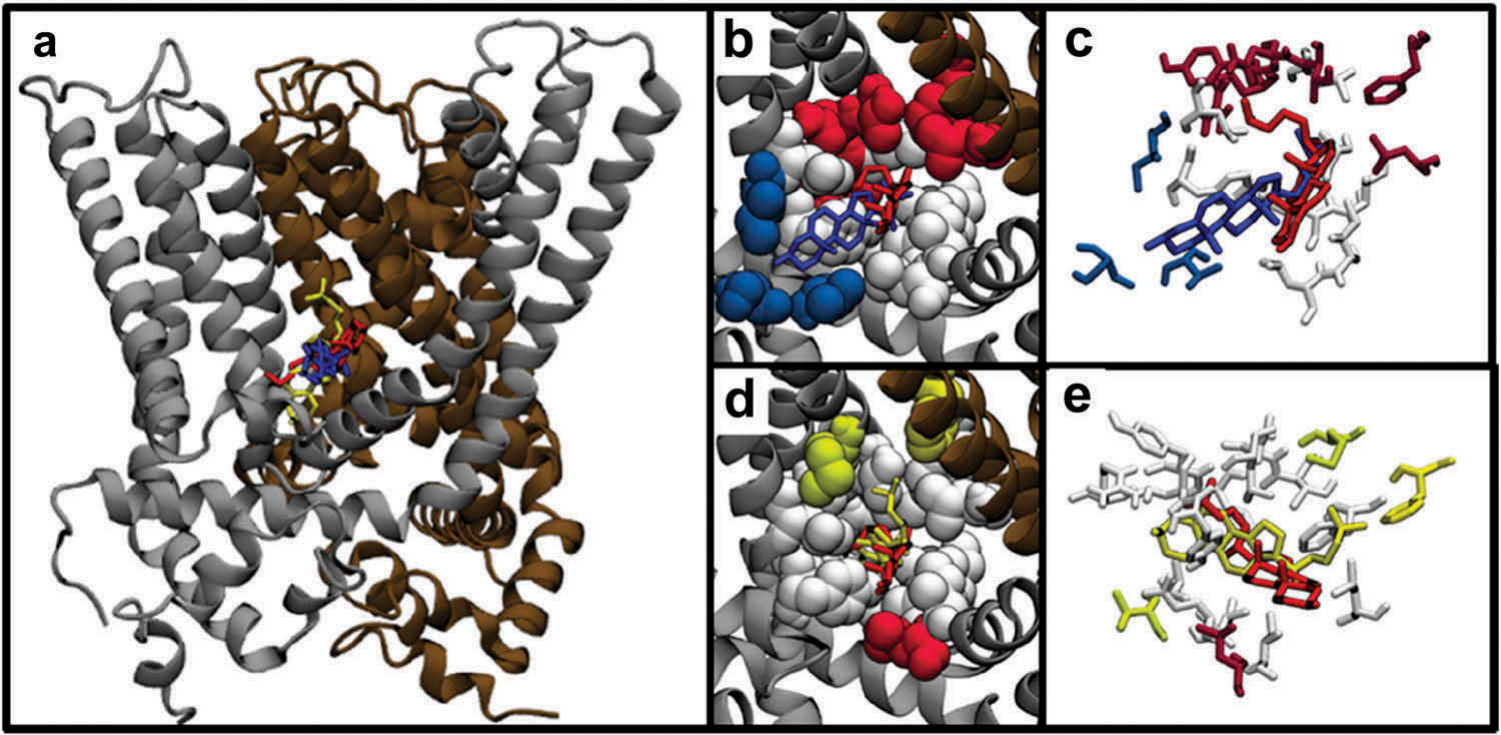
(a) Predicted binding poses of cholesterol, epicholesterol, and ent-cholesterol on the TRPV1, showing a similar predicted binding site, but drastically different binding poses. (b) Comparison of the binding poses of cholesterol and ent-cholesterol. (c) Close-up of the specific residues predicted to interact with cholesterol and ent-cholesterol. (d) Comparison of the binding poses of cholesterol and epicholesterol. (e) Close-up of the specific residues predicted to interact with cholesterol and epicholesterol.

As with Kir2.2 and KirBac1.1, docking analyses were performed on the transmembrane region of TRPV1. The top scoring poses for cholesterol, epicholesterol, and ent-cholesterol were compared, and similarly to what we found in Kir channels, here we also find that all three isomers are predicted to bind to the channel in a similar location. The top scoring poses of all three isomers shared nearly identical average predicted binding energies: −8.3, −8.3, and −8.36 kcal/mol, respectively. Furthermore, these energies are comparable to the binding energies predicted for Kir2.2 and KirBac1.1. There are also some notable differences: docking analyses predicted binding poses within a similar pocket on the channel, but with different orientations, more different than those in the Kir channels (Figure 3(a)). Unlike with Kir channels, where the sterol rings overlapped and the main difference in orientations was due to opposite facing methyl groups, here the sterol rings are oriented more orthogonally to one another. These differences in orientation are reflected in their RMSD scores, which are 8.81 Å and 6.50 Å for epicholesterol and ent-cholesterol, much higher than either the scores for KirBac1.1 or Kir2.2. Overall, there are seven residues predicted to interact with all three sterols, Tyr^131^, Leu^135^, Leu^173^, Ala^186^, Ile^189^, Glu^190^, and Ile^193^. However, due to the differences in poses, these residues are predicted to interact with different regions of each isomer. Likewise, compared to either of the Kir channels, there are many more residue interactions unique to only one or two of the isomers. In particular, there are seven residues that are predicted to interact with cholesterol, but not ent-cholesterol (Ser^132^, Thr^170^, Asn^171^, Tyr^174^, Arg^177^, Phe^207^, and Leu^266^), and a single residue, Leu^194^, that is predicted to interact with cholesterol but not epicholesterol. A detailed representation of all the isomer-specific interactions can be seen in the Venn diagram in **Suppl. Fig. 2B**.

### GABA_A_ and BK predict partial overlap of cholesterol isomers

**Figure 4.**
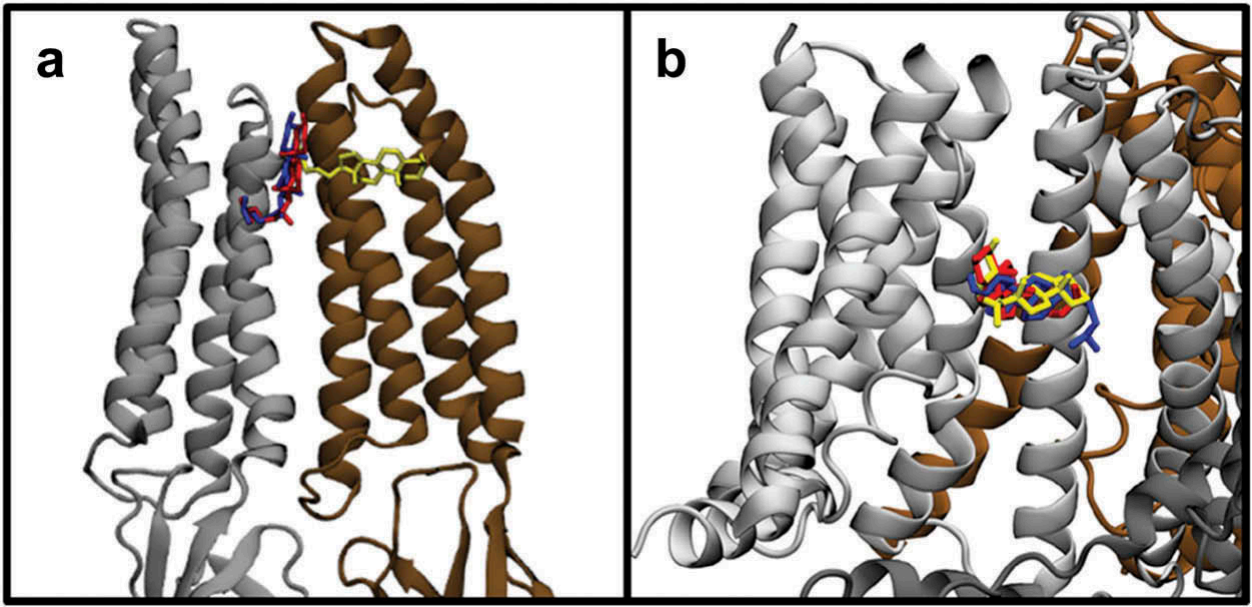
(a) Predicted binding poses of cholesterol (red), epicholesterol (yellow), and ent-cholesterol (blue) on the GABA_A_ channel. Two sterols, cholesterol and ent-cholesterol, show overlap in predicted binding location, while epicholesterol shows partial overlap. (b) Predicted binding poses of cholesterol (red), epicholesterol (yellow), and ent-cholesterol (blue) on the BK channel.

**Figure 5.**
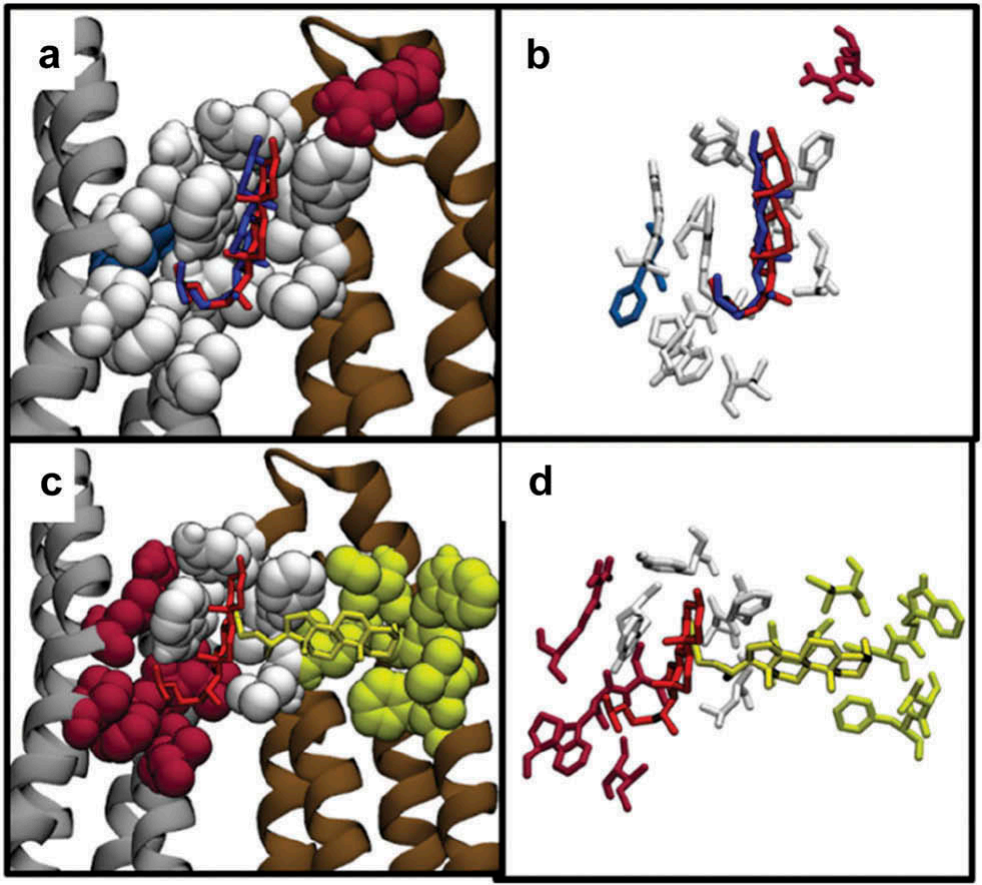
(a) Comparison of the predicted binding poses of cholesterol and ent-cholesterol on GABA_A_. (b) Close-up of the specific GABA_A_ residues predicted to interact with cholesterol and ent-cholesterol. (c) Comparison of the predicted binding poses of cholesterol and epicholesterol on the GABA_A_. (d) Close-up of the specific GABA_A_ residues predicted to interact with cholesterol and epicholesterol.

Docking analyses were also performed for cholesterol, epicholesterol, and ent-cholesterol on the GABA_A_ channel and the BK channel, two other channels for which cholesterol stereospecificity was shown experimentally [6,21]. As with TRPV1 and the Kir channels, we found that all three sterols were predicted to bind to GABA_A_ and BK channel, and with comparable binding energies: −8.1, −8.4, and −9.3 kcal/mol for GABA_A_ and −7.53, −7.47, and −7.0 kcal/mol for BK channel. Likewise, similar to TRPV1, there is overlap in the predicted binding site for cholesterol and its stereoisomers on both channels, but with some differences. In particular (Figure 4), one of the sterols in each case shows partial overlap with the other two sterols. As can be seen in Figure 4(a), the predicted interaction sites for cholesterol and ent-cholesterol on the GABA_A_ receptor overlap closely, while the binding site for epicholesterol partially overlaps. On the BK channel, the predicted binding site for cholesterol, ent-cholesterol, and epicholesterol overlap, but the orientation of epicholesterol is anti-parallel to cholesterol. These positions are reflected in the calculated RMSD scores: 3.29Å and 9.72Å, and 8.62Å and 4.0Å for GABA_A_ and BK, respectively. This partial overlap of binding sites can also be seen in specific residues interacting with each sterol on GABA_A_ (Figure 5(a-d)) and BK channel (Figure 5(e-h)).

#### GABA_A_

Similar to Kir2.2 and KirBac1.1, the predicted binding sites for cholesterol and ent-cholesterol on the GABA_A_ receptor show a high degree of overlap. The predicted poses for each isomer are oriented such that their carbon rings are overlapping, with their methyl groups oriented in opposite directions. Consequently, the list of residues they are predicted to interact with nearly overlap, with both isomers sharing Ile^234^, Trp^237^, Val^238^, Arg^428^ Leu^297^, Ala^300^, Phe^301^, Tyr^304^, Arg^428^, and Pro^432^. Additionally, cholesterol is predicted to interact with Arg^312^, while ent-cholesterol is predicted to interact with Phe^240^. In contrast, the predicted binding sites for cholesterol and epicholesterol, which have a partial overlap, share the residues Trp^241^, Leu^297^, Ala^300^, Phe^301^, and Tyr^304^, while residues Ile^234^, Trp^237^, Val^238^, Arg^428^, and Pro^432^ are unique to cholesterol and residues Glu^298^, Ile^423^, Trp^426^, Ser^427^, Val^430^, and Phe^431^ are unique to epicholesterol (Figure 5(a,b)). Furthermore, the overlapping residues are predicted to interact with very different portions of each sterol. While these residues are predicted to interact near the hydroxyl group for cholesterol, they are predicted to interact with the tail group of epicholesterol.

#### BK channel

**Figure 6.**
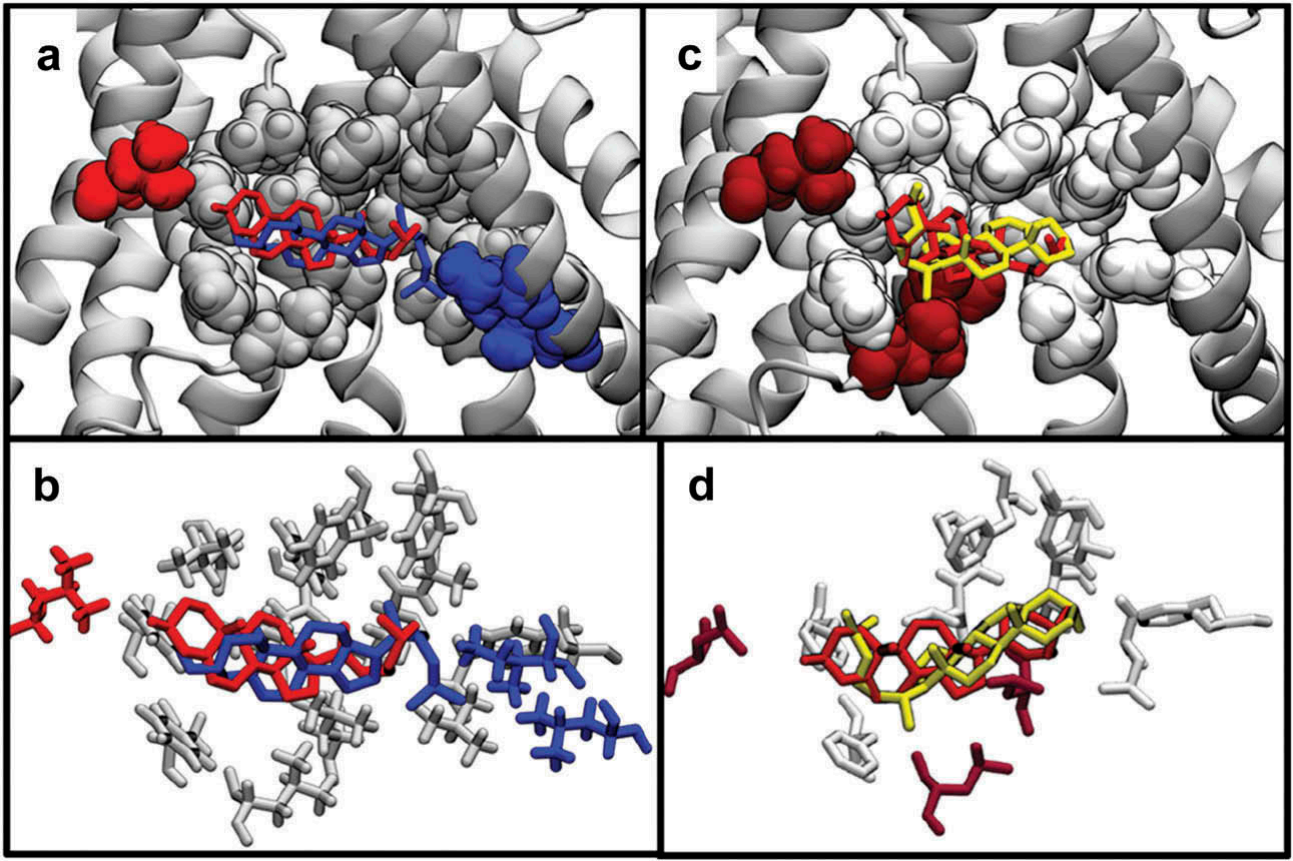
(a) Comparison of the predicted binding poses of cholesterol and ent-cholesterol on the BK channel. (b) Close-up of the specific BK channel residues predicted to interact with cholesterol and ent-cholesterol. (c) Comparison of the predicted binding poses of cholesterol and epicholesterol on the BK channel. (d) Comparison of the predicted binding poses of cholesterol and epicholesterol on the BK channel.

The predicted binding sites for the three isomers on the BK channel follow a similar pattern to those on the GABA_A_ receptor, with all isomers being able to occupy a similar site with comparable binding energies listed above (Figure 6). Interestingly, ent-cholesterol can also binding to a different site of BK with slightly higher favorability, which might be important for interpreting experiments where cholesterol effects on BK are compared to the effects of ent-cholesterol. Specifically, cholesterol and ent-cholesterol share residues Leu^170^, Phe^173^, Phe^177^, Phe^195^, Phe^197^, Leu^198^, Leu^201^, Leu^234^, Thr^237^, Ala^238^, Phe^241^, Phe^292^, and Phe^300^, with Leu^24^ unique to cholesterol and Phe^299^ and Leu^303^ unique to ent-cholesterol. Likewise, cholesterol and epicholesterol share residues Phe^173^, Phe^177^, Phe^197^, Leu^198^, Leu^234^, Thr^237^, Ala^238^, Phe^241^, Phe^292^, and Phe^300^, with epicholesterol having no unique residues and cholesterol interacting uniquely with Leu^24^, Leu^170^, and Leu^201^. This is due to the predicted orientations of cholesterol and epicholesterol with respect to one another. Unlike cholesterol and ent-cholesterol, which have hydroxyl groups oriented in similar directions, the epicholesterol is oriented in the opposite direction when compared to cholesterol.

### Residue makeup of predicted binding sites on stereoselective ion channels

**Figure 7.**
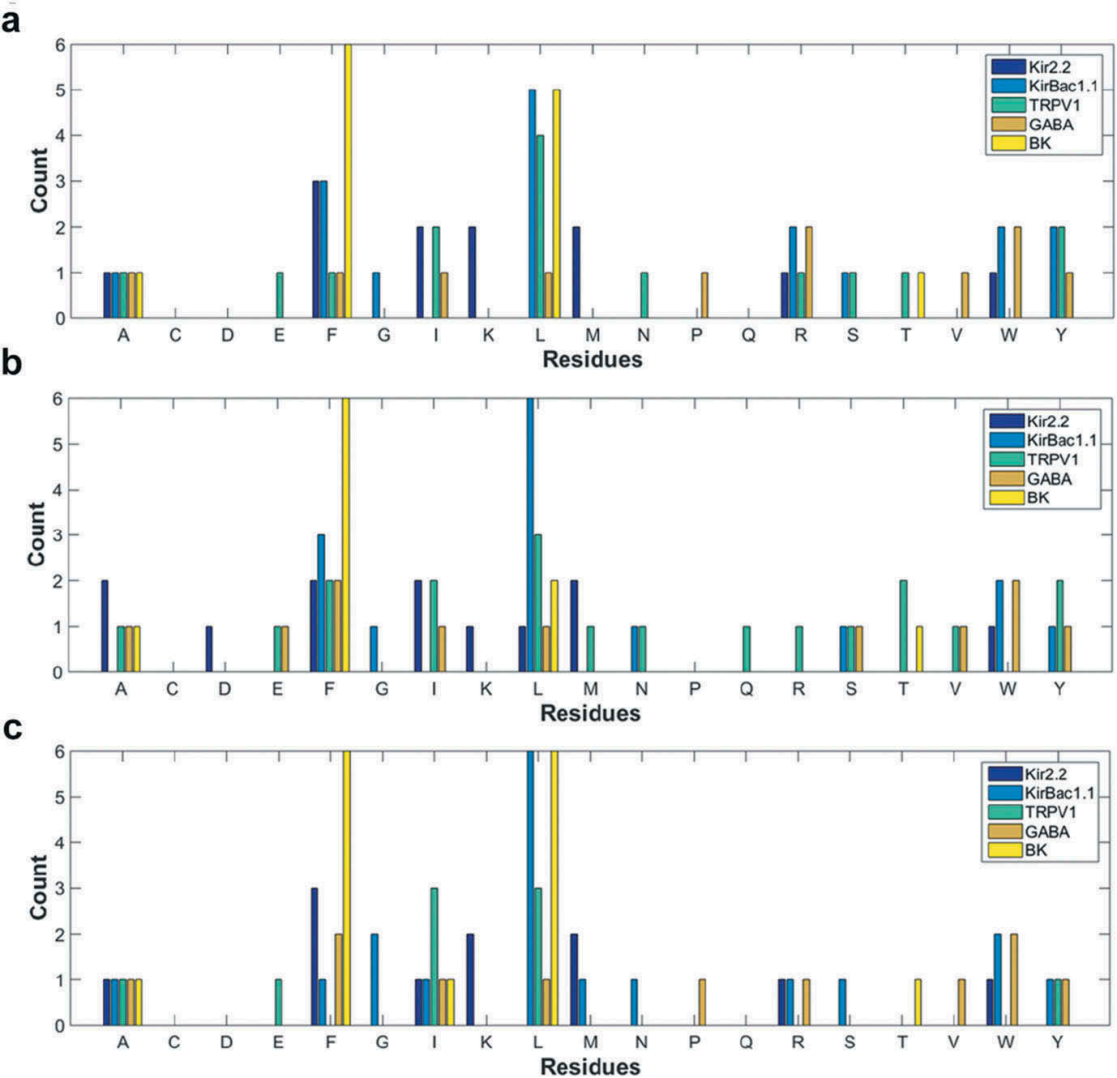
(a) Histogram of different amino acid residues identified in the predicted binding sites for cholesterol in Kir2.2, KirBac1.1, TRPV1, GABA_A_, and BK. (b) Histogram of different amino acid residues identified in the predicted binding sites for epicholesterol in Kir2.2, KirBac1.1, TRPV1, GABA_A_, and BK. (c) Histogram of different amino acid residues identified in the predicted binding sites for ent-cholesterol in Kir2.2, KirBac1.1, TRPV1, GABA_A_, and BK.

In addition to examining the predicted poses and binding energies of cholesterol, epicholesterol, and ent-cholesterol, we also quantified the differences in the types of predicted interacting residues (Figure 7). We found that for all three sterols, hydrophobic residues account for ~60–90% of the binding sites on the different ion channels, with aliphatic residues such as alanine, leucine, and isoleucine appearing in almost every site. Likewise, phenylalanine occurs frequently, being absent only from the ent-cholesterol-binding sites of TRPV1 and the BK channel. No predicted binding sites on any of the channels or for any of the sterols contained a cysteine, and very few contained an Asn or Gln residue, with Gln appearing in all three sites of TRPV1, and the epicholesterol-binding site of GABA_A_, and Asn only appearing in the epicholesterol-binding site of Kir2.2 and the ent-cholesterol-binding site on BK. For a given ion channel, there are some differences in the total numbers of residues comprising bindings sites for the different chiral isomers. For KirBac1.1, there are respectively 17, 15, and 18 residues that make up the cholesterol, epicholesteorl, and ent-cholesterol-binding sites. For TRPV1, there are 15, 19, and 9 residues. For GABA_A_ and BK there are 11, 11, 11, and 13, 10, and 15 respectively.

## Discussion

The main finding of this study is that chiral isomers of cholesterol dock to several types of ion channel proteins in the same putative sites as the cholesterol molecule. This observation suggests that cholesterol binding to these channels exhibits only partial stereospecificity with only minor differences between favorable binding configurations and comparable binding energies. This conclusion is in contrast to the previous belief that chiral isomers of cholesterol do not bind to cholesterol-binding sites of membrane proteins in general and ion channels in particular. The significance of this new finding is that it suggests a new paradigm for the nature of the stereospecificity of cholesterol regulatory effects on ion channel activity. Previously, since cholesterol and its chiral analogs were shown to have differential effects on ion channel function, it was generally assumed that a lack of the functional response of cholesterol isomers was due to a lack of binding but comparative docking analysis presented in this study suggests that this is not the case. Previous studies showed that for all the channels explored in this study, the channels discriminate between cholesterol and its chiral analogs. Specifically, while cholesterol suppresses the activities of Kir, BK and TRPV1 channels, epicholesterol and ent-cholesterol may have an opposite or no effect. Similarly, for GABA, while cholesterol supports the function of the channels, epicholesterol does not. Our analysis predicts, however, that the differential functional effect of cholesterol on the channel relative to its stereoisomers results not from a lack of binding, but from the specific structural arrangement and its interactions of the cholesterol molecule within its binding site. This prediction supports our previous analysis of cholesterol interaction with Kir2 channels [20]. Here, we show that the same principle appears to apply to several types of structurally unrelated ion channels.

The current analysis is based on one of the most established computational approaches to identify the potential binding sites of a variety of ligands to proteins and characterize the properties of these interactions [24–26]. We and others have previously used docking analyses to predict the binding sites of cholesterol on Kir2.1 [27] and Kir2.2 [16,20]. In both cases, the predictions of the docking analysis were validated by site-directed mutagenesis, followed by electrophysiological recordings. Likewise, docking analyses were used to predict cholesterol-binding sites on a number of ion channels, including TRPV1 and GABA_A_ [18,28]. Similar to previous studies, a version of the AutoDock program was used to perform these docking analyses. In the current study we employed the most recent version of the software, AutoDock Vina, which was demonstrated to be both faster and more accurate than previous iterations of the program [22]. Furthermore, since an increasing number of transmembrane proteins have been co-crystallized with cholesterol, we could validate our approach by docking cholesterol to a protein with a known cholesterol-binding site. Specifically, we chose the cholesterol-binding site on the β2-adrenergic receptor, one of the well characterized cholesterol-binding sites that was confirmed by crystal structure [29,30]. As expected, we found that the most favorable AutoDock-predicted pose strongly overlapped with the crystal structure. This observation supports the notion that our docking approach was sufficient to predict an already known cholesterol-binding site identified through crystallography. Interestingly, a recent study revealed a solved crystal structure of TRPC4 channel with a bound cholesteryl hemisuccinate, a modified cholesterol molecule [31]. However, since the structure of cholesteryl hemisuccinate is significantly different from that of cholesterol, featuring a succinate group instead of a hydroxyl group, we did not perform a docking analysis on TRPC4.

It is also important to recognize however that both the resolution of the available crystal structures for the ion channel proteins explored in this study and the docking analysis itself have limitations. As described in the Methods section, the resolutions for all the structures was ~3A, which is recognized to provide the contours of the protein with the atomic structure being inferred (https://pdb101.rcsb.org/learn/guide-to-understanding-pdb-data/resolution). Nevertheless, multiple studies used these crystal structures to obtain significant insights into the ligand binding to the channels [16,32,33]. Another important limitations is that docking analyses typically use a static protein structure and employ molecular force fields or turnkey methods for predicting favorable binding poses and energies, which by nature are an approximation [25,34]. The use of static structures is a major limitation, as proteins in their native environment can have flexibility in their secondary and tertiary structures. Specifically, for binding events, amino acids in a binding pocket can rotate and shift position to accommodate different ligands [35], something which is not taken into account in a static-structure docking study. An example of this would be inter-subunit binding sites, wherein a cholesterol molecule inserts itself and shifts the surrounding structure. This limitation can be overcome through the use of molecular dynamics simulations, which use the docked pose of the ligand as a starting point to better study these dynamics, as we and others did for a variety of proteins [e.g. 16, 27, 28]. This approach however is unfeasible for a comparative study that includes multiple sterols binding to multiple proteins, as is done in the current study. However, even with these limitations, docking analyses are a powerful approach to provide initial insights and screening into potential cholesterol-binding sites.

It is also important to compare the predictions for cholesterol-binding sites obtained in the current study with previously reported based on docking and simulations. As expected, the docking analysis for Kir2.2 yielded similar results to previous studies from us and others for Kir2.1 and Kir2.2 [16,27]. A very similar site was also found for cholesterol on KirBac1.1, with cholesterol oriented in the same direction as on Kir2.2 and in an equivalent location near the lipid membrane interface. The predicted binding site on TRPV1 represents a similar but not identical site to what was identified previously. This is not surprising however, as the previous prediction was made before a crystal structure of TRPV1 was discovered and used a homology model of TRPV1 [18]. Likewise, the same is true for GABA_A_ [28]. Thus overall, our current predictions for cholesterol-binding sites described above are consistent with previous studies. The ability of the channels to accommodate cholesterol and their analogs was observed regardless of whether the docking analysis was performed for the open state of the channels (Kir2.2, BK) or the closed state (KirBac1.1, TRPV1, GABA). A more detailed analysis may uncover some differences between the conformation states of the same channel but this is beyond the scope of the current study. The main novelty of our observations is that these cholesterol-binding sites are not stereospecific, and that both cholesterol analogs, epicholesterol and ent-cholesterol, are predicted to bind the same sites with comparable binding energies in all the channels and all the conformations tested in this study. As described above, this was most surprising because these channels exhibit stereospecificity in their functional response to cholesterol, and the prevailing assumption was that this stereospecificity was due to a lack of binding.

An attractive idea is that a lack of chiral specificity of cholesterol binding can be related to our recent computational work, which showed that a Kir2.2 channel simultaneously interacts with an ensemble of cholesterol molecules, which could interact with the channel at multiple different sites [36]. Furthermore, bound cholesterol molecules were found to be highly flexible within their sites, in part due to the fact that bound molecules were stabilized by hydrophobic interactions, rather than hydrogen bonds. This phenomena causes cholesterol to behave differently than more traditional “drug-like” ligand molecules, which tightly bind to their target receptors through multiple hydrogen bonds, as discussed in several recent studies [36–38]. It is also interesting to note that recent atomistic simulations of the β2-adrenergic receptor found that specific oxysterols and cholesterol analogs such as cholesteryl hemisuccinate, 4β-OH-cholesterol and 27-hydroxycholesterol, are also capable of binding to the same binding sites as cholesterol, and compete for these binding sites when present together [39]. However in the case of the β2-adrenergic receptor, it is not known whether cholesterol analogs bound to the same sites elicit differential functional responses, as was shown for the ion channels explored in our current study. This is a very important point, because if stereoisomers bind to the same sites and elicit the same responses, it means that the site is not discriminating between the stereoisomers. In contrast, if stereoisomers bind to similar sites but elicit significantly different responses, it means that the functional interactions of the isomers within the site are of utmost significance.

In summary, based on our docking analyses, we propose that the stereospecificity of ion channels is not caused by the binding of cholesterol, but rather by the specific residue interactions of cholesterol within its binding site, relative to its chiral isomers. In all the ion channels tested, we observed specific amino acid residues that were predicted to interact only with cholesterol, or predicted to interact with cholesterol in a unique manner when compared to epicholesterol or ent-cholesterol. We propose that analysis of these unique residue interactions would provide major insights into the nature of cholesterol regulation of ion channels.
