# Supplementary material for: Comparative docking analysis of cholesterol analogs to ion channels to discriminate between stereospecific binding vs. stereospecific response
Source: Channels (Austin). 2019 Apr 29;13(1):136–46. doi: 10.1080/19336950.2019.1606670 (PMC6527060; doi:10.1080/19336950.2019.1606670)

Supplemental Figure 1. Example Configuration File

```
receptor = 3SPI.pdbqt
ligand = cholesterol.pdbqt

exhaustiveness 40

num_modes 20

center_x = -55.333
center_y = -28.762
center_z = 45.464

size_x = 32.25
size_y = 33
size_z = 42.75

out = Kir2_Cholesterol.pdbqt
```

Supplemental Figure 2. Venn Diagrams showing overlap of interacting residues for **A)** Kir2.2 **B)** KirBac1.1 **C)** TRPV1 **D)** $GABA_A$ **E)** BK, with Cholesterol (red), ent-cholesterol (blue), and epicholesterol (yellow).

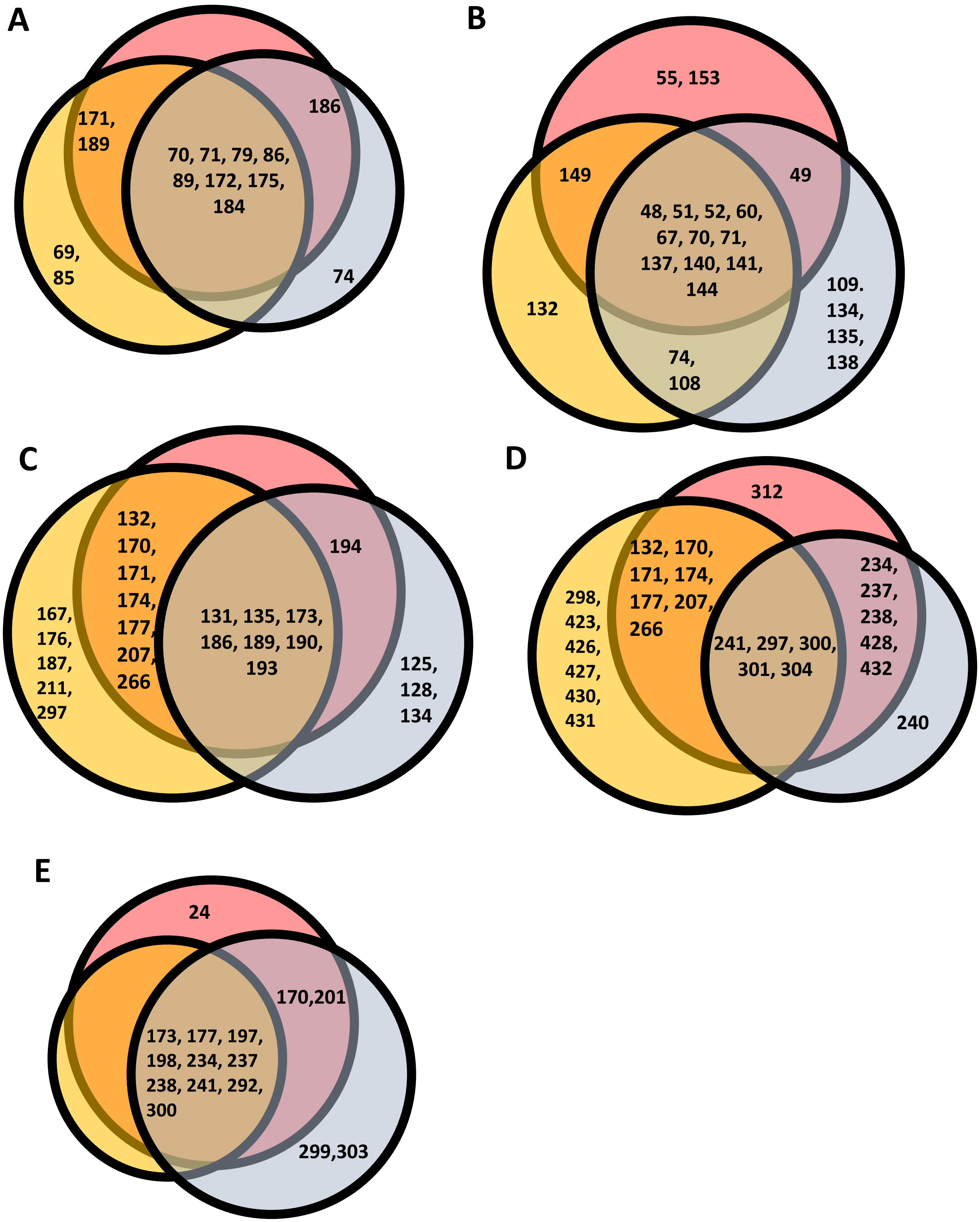

Supplement: Supplemental Material [file kchl-13-01-1606670-s001.pdf]
